# Fragment Enrichment of Circulating Tumor DNA With Low-Frequency Mutations

**DOI:** 10.3389/fgene.2020.00147

**Published:** 2020-02-28

**Authors:** Xiaojun Liu, Jidong Lang, Shijun Li, Yuehua Wang, Lihong Peng, Weitao Wang, Yingmin Han, Cuixiao Qi, Lei Song, Shuangshuang Yang, Kaixin Zhang, Guoliang Zang, Hong Pei, Qingqing Lu, Yonggang Peng, Shuxue Xi, Weiwei Wang, Dawei Yuan, Pingping Bing, Liqian Zhou, Geng Tian

**Affiliations:** ^1^ School of Computer Science, Hunan University of Technology, Zhuzhou, China; ^2^ Bioinformatics Department, Geneis (Beijing) Co. Ltd., Beijing, China; ^3^ Department of Pathology, Chifeng Municipal Hospital, Chifeng, China; ^4^ Academics Working Station, Changsha Medical University, Changsha, China

**Keywords:** low-frequency tumor mutation, cell-free DNA, circulating tumor-derived DNA, fragment length enrichment, mutant allele frequency, next generation sequencing

## Abstract

Human blood contains cell-free DNA (cfDNA), with circulating tumor-derived DNAs (ctDNAs) widely used in cancer diagnosis and treatment. However, it is still difficult to efficiently and accurately identify and distinguish specific ctDNAs from normal cfDNA in cancer patient blood samples. In this study, ctDNA fragment length distribution analysis showed that ctDNA fragments are frequently shorter than the normal cfDNAs, which is consistent with previous findings. Interestingly, the ctDNA fragment length was found to be partially associated with the mutant allele frequency, with a low mutant allele frequency (< ~0.6%) associated with a longer ctDNA fragment length when compared to normal cfDNAs. The findings of this study contribute to improving the detection of low-frequency tumor mutations.

## Introduction

In modern medicine, liquid biopsies are widely used in prenatal diagnoses and cancer treatment. When utilizing a liquid biopsy, circulating cell-free DNA (cfDNA), circulating tumor cells (CTCs), or exosomes are isolated for evaluation (Bardelli and Pantel, 2017; Wan et al., 2017; Siravegna et al., 2017). Of these, circulating tumor-derived DNA (ctDNA) is widely utilized as a tumor biomarker in translational and clinical research (Diaz and Bardelli, 2014; Donaldson and Park, 2018), while fetal cfDNA obtained from maternal blood is widely used as a noninvasive method for prenatal diagnoses (Lun et al., 2008; Lo et al., 2010; Yu et al., 2014; Sun et al., 2018).

About 30 years ago, Stroun et al. first discovered that cancer patient blood samples contain cfDNA of cancer origin (Stroun et al., 1989; Thierry et al., 2016). In the following decades, ctDNA has been gradually developed as a clinical tool for cancer diagnosis and treatment, and has even been used as a prognostic or predictive factor (Mao et al., 1994; Lecomte et al., 2002; Kimura, 2006; Diehl et al., 2008). Currently, the use of ctDNA detection in cancer therapy has been approved by the US Food and Drug Administration as a treatment determinant (osimertinib or erlotinib) in non-small-cell lung carcinoma (NSCLC) patients with an *EGFR* mutation in the event that a tumor biopsy cannot be performed (US Food & Drug Administration, 2016). The application of ctDNA in cancer therapy is reliant on precise polymerase chain reaction (PCR)-based technologies, such as droplet digital PCR (ddPCR) or amplification refractory mutation system (ARMS)-PCR, and deep-sequencing technologies; these techniques aid in distinguishing ctDNAs from other normal cfDNAs within the plasma and enable hotspot mutation detection within cancer driver genes (Taly et al., 2013; Newman et al., 2014; Frenel et al., 2015; Azizi et al., 2018). However, ctDNAs are usually present in low abundance relative to the normally occurring cfDNAs derived from normal cells, particularly in non-metastatic solid tumors (Tug et al., 2014; Siravegna et al., 2017). Consequently, there is an urgent need to reliably distinguish ctDNAs from normal cfDNA to improve the accuracy of identifying driver gene mutations.

Recently, tumor-derived ctDNAs have been shown to vary in size and are shorter than normal cfDNAs in healthy people (Umetani et al., 2006; Thierry et al., 2010; Mouliere et al., 2011; Mouliere et al., 2013). This trend was also observed during pregnancy, with fetal cfDNA usually of a different fragment size than the maternal cfDNA (Lun et al., 2008; Lo et al., 2010). Furthermore, in one study examining ctDNA length distributions in hepatocellular carcinoma (HCC) patients, copy number aberrations were leveraged and showed that high-concentration ctDNA fractions were more fragmented, while low-concentration fractions were paradoxically longer (Jiang et al., 2015; Mouliere and Rosenfeld, 2015; Jiang and Lo, 2016). In another study, ctDNAs were found to be consistently shorter than normal cfDNA, in both animal xenograft models and clinical plasma samples (Underhill et al., 2016). Additionally, mutant ctDNA fragments from tumor patients were always shorter than wild-type cfDNA fragments from healthy donors, with mutant alleles more commonly having shorter fragment lengths, something that could potentially be exploited to improve ctDNA detection (Underhill et al., 2016; Hellwig et al., 2018). Moreover, a later study confirmed that this size difference could be exploited to enhance sensitivity when monitoring ctDNAs and for noninvasive genomic analysis of various cancers (Mouliere et al., 2018). However, few studies have examined the impact of mutant allele frequency on the size distribution of ctDNA fragments, and most studies were conducted in cancer patients with relatively high mutant allele frequencies.

Thus, the aim of this study was to examine ctDNA fragment distributions in patients with low mutant allele frequencies and determine whether the ctDNA fragment length is affected by the mutant allele frequency. This was accomplished by utilizing blood samples from cancer patients with a variety of different histological types and stages. Key driver gene mutation frequencies were determined using deep-sequencing technologies and ddPCR, and fragment length differences between mutant ctDNAs and normal cfDNAs obtained from the cancer patient samples were examined.

## Materials and Methods

### Sample Collection

All 105 samples (male: 49.52%, female: 50.48%) were obtained from lung cancer patients from Chifeng Municipal Hospital. All patients provided informed written consent before de-identification. The median age of the patients was 63.5 years old (range from 36 to 85 years old). Our research was approved by the Medical Research Ethics Committee of Chifeng Municipal Hospital (Ethics [2018] No. 017).

### Next-Generation Sequencing (NGS) Library Preparation, Sequencing, and Bioinformatics

Cell-free DNA was extracted using a QIAamp Circulating Nucleic Acid Kit (Qiagen, Hilden, Germany) according to the manufacturer’s instructions. The extracted DNA (20 ng/sample) was then used to build libraries using Accel-NGS^®^ 2S Plus DNA Library Kits (96 reactions; Swift BioSciences, Ann Arbor, MI, USA). Customized probes were obtained from Integrated DNA Technologies (IDT, Skokie, IL, USA) and were used for hybridization capture. All cfDNA libraries utilized a 38-hotspot gene panel (**Supplementary File**) and were quantified using a Universal Library Quantification Kit (Kapa Biosystems, Wilmington, MA, USA) on an ABI 7500 Real-Time PCR system (Applied Biosystems, Waltham, MA, USA). Sample quality was evaluated using a high sensitivity DNA kit (Agilent Technologies, Santa Clara, CA, USA) with an Agilent 2100 Bioanalyzer as per the manufacturer’s instructions. NGS with fusion detection was performed using a NextSeq 500/550 High Output v2 kit with a NextSeq 500 sequencer (Illumina, San Diego, CA, USA) for 302 cycles, with standing paired-end reads of 151 bp (average sequencing depth was ~2,164X, details in the **Supplementary File**).

The FASTQ reads were collapsed into unique observations based on barcodes using CASAVA (v1.8.2) software. Low-quality and adapter-contaminated reads were removed from the raw reads using Cutadapt (v1.12) and aligned to the Hg19 reference genome using the Burrow-Wheeler Aligner for short-read alignment (bwa aln; 0.7.12-r1039). Paired-end reads with hotspots were extracted from the paired-end alignment information (column 9th) in BAM format using Samtools (v0.1.19-44428cd), and the corresponding insert size information was extracted. Finally, the extracted paired-end reads were aligned to the Hg19 reference genome again using SOAP (2.21), and hotspot mutation fragment lengths and wild fragment lengths were calculated with the alignment mismatch information (column 11th) in the alignment files.

### Digital Droplet PCR

*EGFR*-T790M, *EGFR*-L858R, *BRAF*-V600E, *PIK3CA*-E545K, *KRAS*-G12C, and *KRAS*-G12V mutant allele frequencies were determined using a Digital Droplet PCR system (Bio-Rad Laboratories, Inc., Hercules, CA, USA), with a droplet size of 1 nL in a total reaction volume of 20 μL, with ~20 ng of cfDNA library utilized. All primers and probes were synthesized by IDT (Skokie, IL, USA; **Table 1**). Droplet counts were determined using the QuantaSoft software (Bio-Rad).

**Table 1:** Primers and probes used in droplet digital PCR experiments.

Mutation		Forward primer	Reverse primer	Wild probe	Mutation probe
*T790M*		GCCTGCTGGGCATCTG	TCTTTGTGTTCCCGGACATAGTC	VIC-ATGAGCTGCGTGATGAG-MGB-NFQ	FAM-ATGAGCTGCATGATGAG-MGB-NFQ
*L858R*		GCAGCATGTCAAGATCACAGATT	CCTCCTTCTGCATGGTATTCTTTCT	VIC-AGTTTGGCCAGCCCAA-MGB-NFQ	FAM-AGTTTGGCCCGCCCAA-MGB-NFQ
*V600E*		CATGAAGACCTCACAGTAAAAATAGGTGAT	TGGGACCCACTCCATCGA	VIC-CTAGCTACAGTGAAATC-MGB-NFQ	FAM-TAGCTACAGAGAAATC-MGB-NFQ
*E545K*		CACTTACCTCTGACTCCATAGAAAATCTT	AAAGCAATTACTACACGATATCCTCTCTC	HEX-TCCTGCTCAGTGATT-MGB-NFQ	FAM-CTCCTGCTTAGTGATT-MGB-NFQ
*G12*	*G12V*	AATTAGATGTATCGTCAAGGCACTCTT	GCTGAAAATGACTGAATATAAACTTGTGG	VIC-TACGCCACCAGCTC-MGB-NFQ	FAM-TACGCCAACAGCTC-MGB-NFQ
	*G12C*				FAM-TACGCCACAAGCTCT-MGB-NFQ

For the *PIK3CA*-E545K (n = 1), *KRAS*-G12C (n = 3), *KRAS*-G12V (n = 1), *EGFR*-T790M (n = 5), and *EGFR*-L858R (n = 1) samples, amplified libraries were utilized prior to size selection to define gates for wild-type and mutation droplet populations. Libraries were constructed using the obtained DNA (~20 ng) and a Rapid DNA Lib Prep kit (ABclonal, Woburn, MA, USA). The obtained libraries (~1.2 mg) were then separated using 2% agarose gel electrophoresis and bands between 130–160 bp and 160–230 bp were extracted using a QIAquick Gel Extraction kit (Qiagen). All of the selected fragment size libraries were then validated using ddPCR as described above (**Supplementary File**).

## Results

### Comparison of cfDNA Fragment Sizes in Cancer Patient Plasma Samples

**Figure 1:**
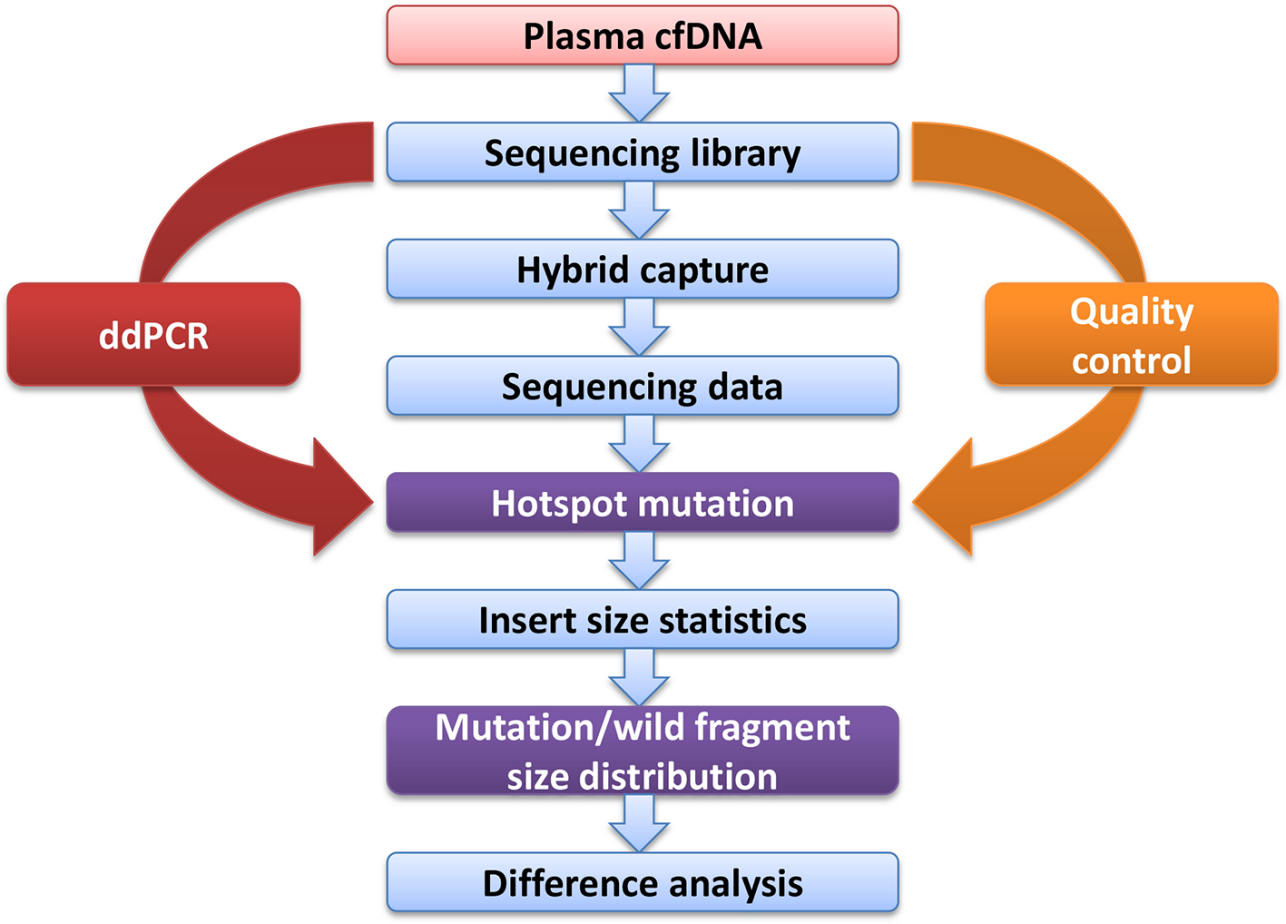
Experimental design flow.

Blood samples were obtained from cancer patients with defined driver gene hotspot mutations, including *EGFR*-T790M (n = 32), *EGFR*-L858R (n = 28), *BRAF*-V600E (n = 13), *PI3KCA*-E545K (n = 13), *KRAS*-G12C (n = 13), and *KRAS*-G12V (n = 6). The cfDNA-sequencing libraries were analyzed by both NGS and ddPCR to precisely detect the mutant allele frequencies of these six hotspots in each cancer patient (**Figure 1**). Some hotspot mutant allele frequencies were more variable, such as *EGFR*-T790M (0.11–74.75%), *EGFR*-L858R (0.15–35.77%), *PI3KCA*-E545K (0.10–21.67%), and *KRAS*-G12C (0.10–33.81%; **Table 2**). Furthermore, some hotspot allele frequencies within these driver genes were relatively low, including *BRAF*-V600E (0.10–0.30%) and *KRAS*-G12V (0.11–1.26%; **Table 2**), which could be explained by examining samples at different tumor stages and of different histological types collectively. Next, the cfDNA-sequencing libraries were sequenced, and size differences between plasma ctDNA and normal cfDNA were compared.

**Table 2:** Summary of the mutation frequencies based on next generation sequencing.

	*T790M*	*L858R*	*V600E*	*E545K*	*G12C*	*G12V*
Validation library number	32	28	13	13	13	6
Low mutation frequency 0.1–1%	16	11	13	11	4	5
Medium mutation frequency 1–10%	10	12	0	1	7	1
High-mutation frequency 10–100%	6	5	0	1	2	0
Mutation frequency distribution	0.11–74.75%	0.15–35.77%	0.10–0.30%	0.10–21.67%	0.10–33.81%	0.11–1.26%

### Mutant Alleles Have a Shorter Fragment Length Than the Wild-Type Alleles

**Figure 2:**
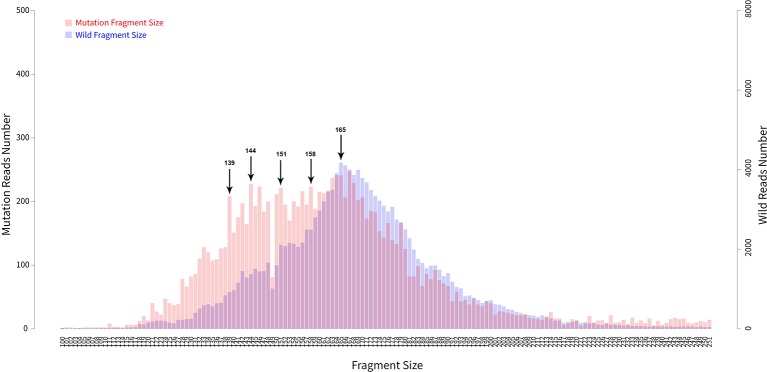
Fragment length distributions of cfDNAs from 105 cancer patient blood samples.

**Figure 3:**
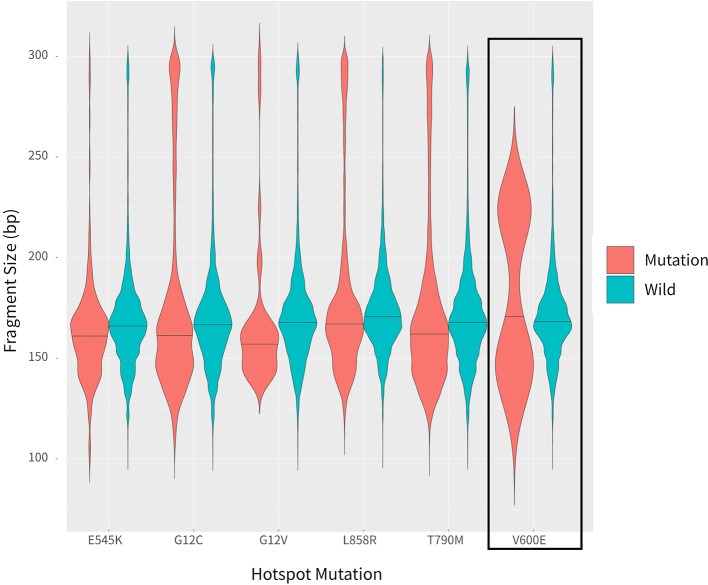
Comparison of fragment length sizes between ctDNAs and normal cfDNAs.

In addition to examining cancer patient mutant allele frequencies, whole cfDNA fragment length distributions were globally observed. As expected, the mutant ctDNA fragments were generally shorter than the normal cfDNAs (**Figures 2** and **3**). In patients with a low mutation frequency, the ctDNA fragment length was longer than the normal cfDNAs, such as *BRAF*-V600E (0.10–0.30%). However, this trend was not observed in the four other DNA fragment size distribution, including *EGFR*-T790M (0.11–74.75%), *EGFR*-L858R (0.15–35.77%), *PI3KCA*-E545K (0.10–21.67%), *KRAS*-G12V (0.11–1.26%) or *KRAS*-G12C (0.10–33.81%; **Figure 3**).

### Longer Fragment Lengths in Mutant ctDNAs With a Low Mutation Frequency

**Figure 4:**
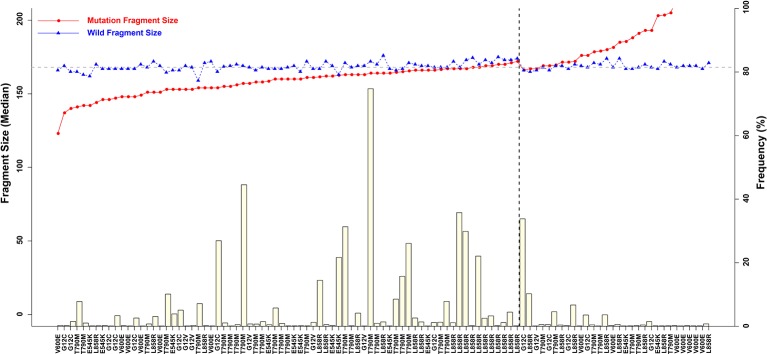
Associations between mutant allele frequency and ctDNA fragment sizes. Gray horizontal dotted line is 168 bp, and black vertical dotted line is L858R.)

**Figure 5:**
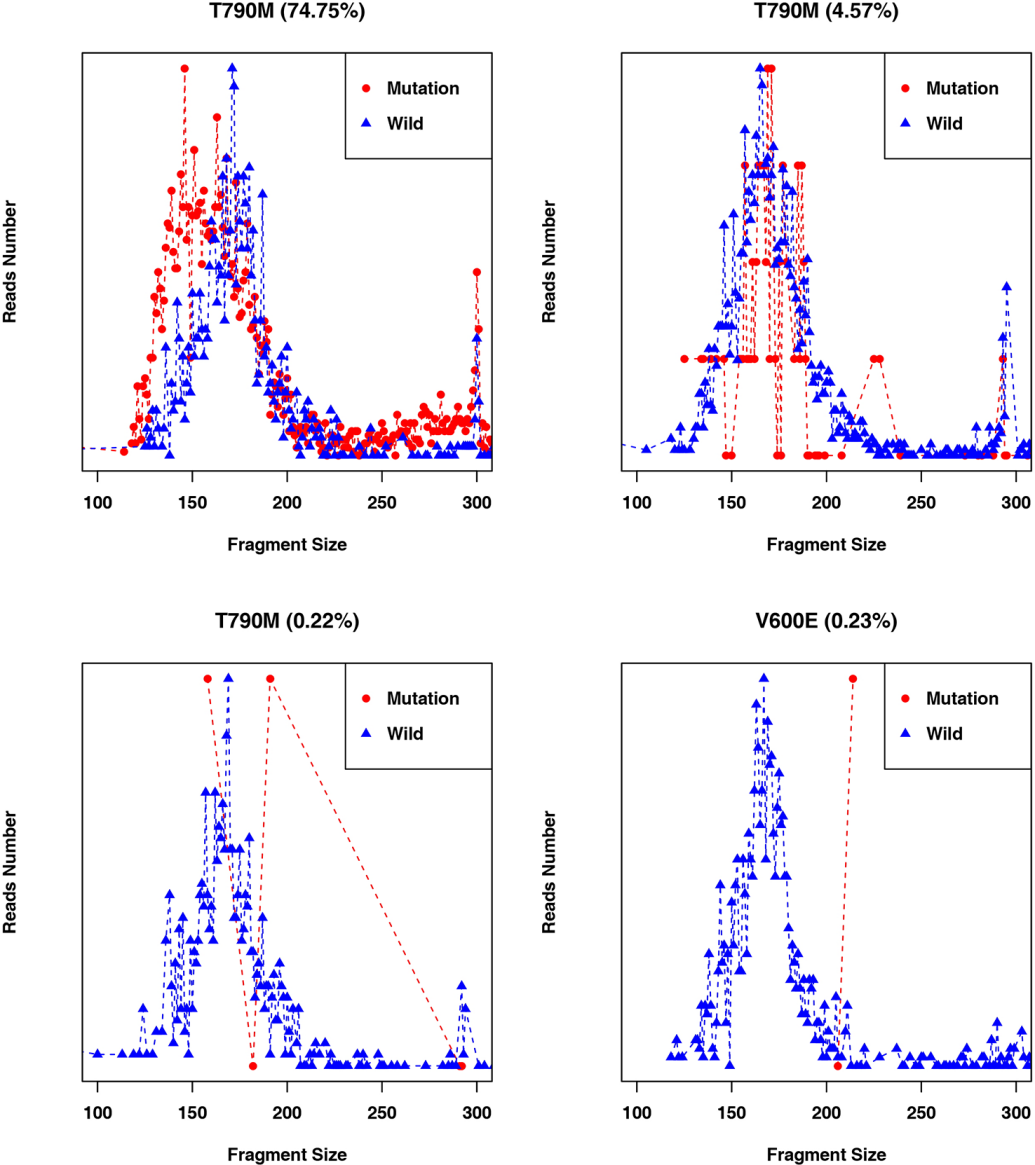
Fragment length distributions of cancer patient ctDNAs and normal cfDNAs with high, medium, or low *EGFR-T790M* mutant allele frequencies.

Fragment size differences between cancer patient ctDNAs and normal cfDNAs were further examined in conjunction with a low, medium, or high mutant allele frequency. In fragments associated with a low mutant allele frequency, the ctDNA fragments were longer than the normal cfDNAs (**Figure 4**), such as *EGFR*-T790M (0.22 and 0.21%). However, in ctDNAs with a higher mutant allele frequency, such as *EGFR*-T790M (74.75%), or a medium frequency, such as *EGFR*-T790M (4.57%), fragment lengths were shorter than the normal cfDNAs (**Figure 5** and **Table 3**).

**Table 3:** Fragment length distributions of cancer patient ctDNAs and normal cfDNAs with high, medium, or low *EGFR-T790M* mutant allele frequencies.

Mutation type	NGS (%)	ddPCR (%)	Description	Mutation peak	Wild peak	Mutation fragment median	Wild fragment median
*T790M*	74.75	69.33	Short	146	171	164	172
*T790M*	4.57	5.55	Long	169/171	165	169.5	169
*T790M*	0.22	0.26	Other	158/191	169	191	168
*V600E*	0.23	0.17	Long	214	167	214	168

### Low-Frequency Mutations Are Associated With Large Fragment Sizes

**Figure 6:**
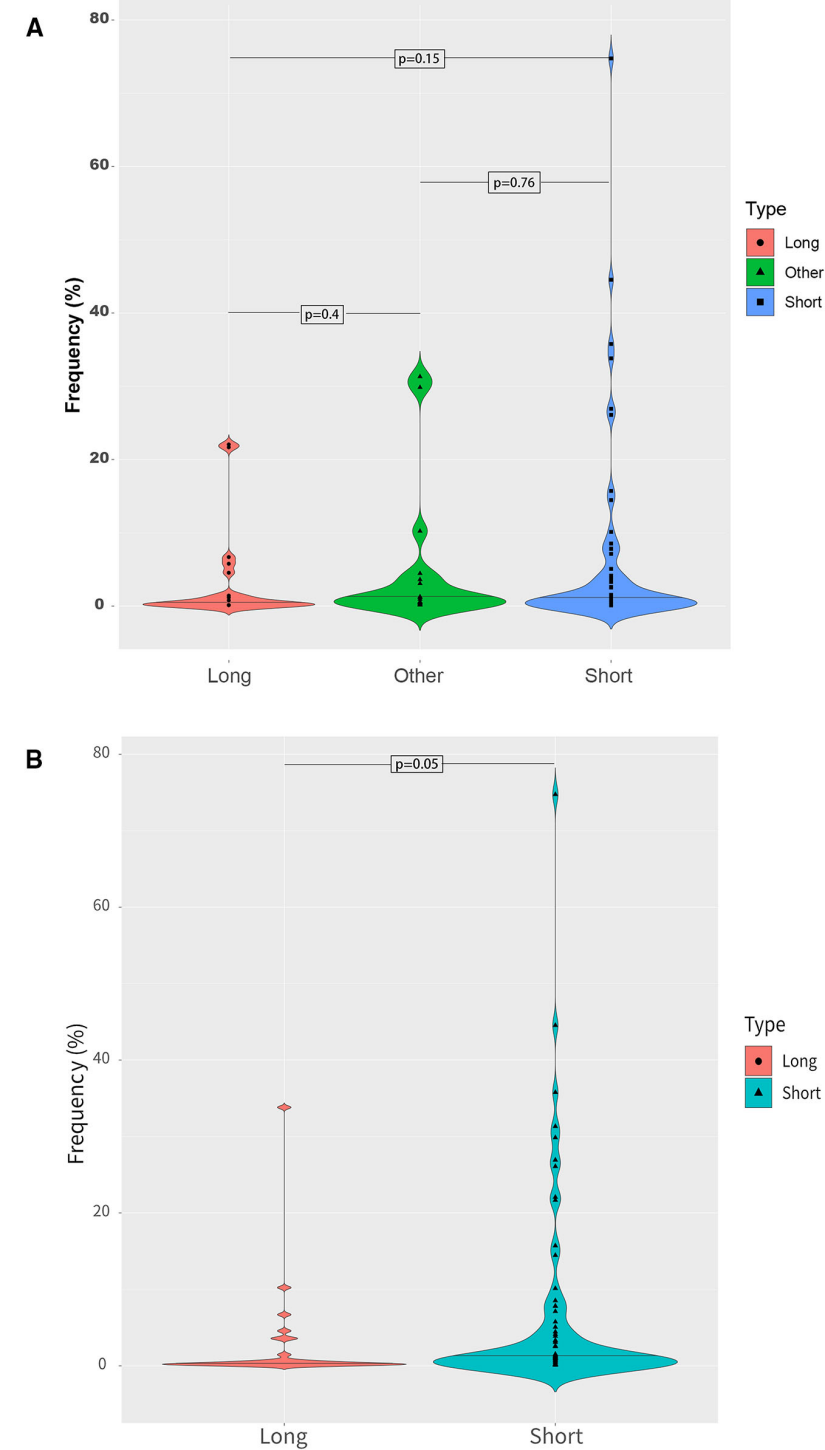
Mutant allele frequency distributions based on ctDNA fragment length. **(A)** Relationship between the mutation fragment size peak and the mutation frequency. **(B)** Relationship between the median mutation fragment size peak and the mutation frequency.

Different fragment sizes were observed among the mutant ctDNAs, including long ctDNA (longer than normal cfDNAs), normal ctDNA (comparable to normal cfDNA lengths), and short ctDNA (shorter than normal cfDNAs). Within these three groups, the mutant allele frequency distributions were examined and showed that a low mutation frequency was commonly associated with a long ctDNA fragment length, while normal and short ctDNAs were not (**Figure 6**).

### Enrichment of Longer ctDNA Fragments Could Improve the Detection of Low-Frequency Mutations

**Figure 7:**
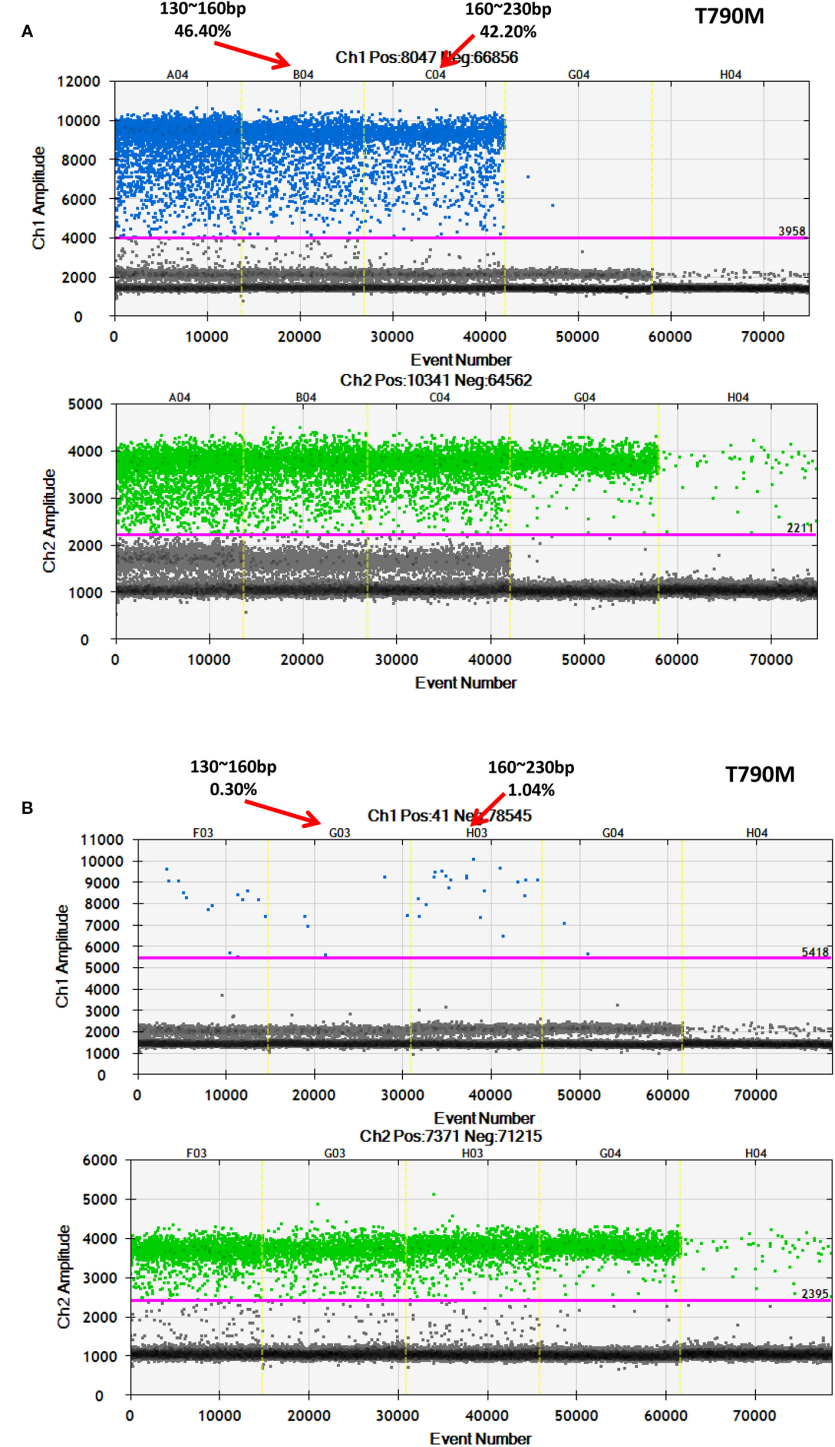
Further validation of an association between fragment size and frequency using ddPCR. Examination of different fragment size libraries from a patient with **(A)** a high *EGFR-T790M* frequency (44.53%) and from a patient with **(B)** a low *EGFR-T790M* frequency (0.54%) using ddPCR.

After discovering that a low-frequency is associated with a longer ctDNA fragment size, this study aimed to determine if enriching longer cfDNA fragments could increase the mutation frequency in blood samples with a low mutant allele frequency. In one patient with a high frequency for *EGFR*-T790M (44.53%), cfDNA was extracted and different fragment sizes were obtained. To further detect the *EGFR*-T790M frequency, DNA libraries comprising two different DNA fragment sizes were examined using ddPCR. The *EGFR*-T790M frequency in a library with a fragment length between 160 and 230 bp (42.20%) was lower than the library with a fragment size between 130–160 bp (46.40%; **Figure 7A**). This was consistent with the findings presented above. Conversely, a cfDNA sample was obtained from a patient with a low *EGFR*-T790M frequency (0.54%) and different fragment sizes were collected and analyzed. In the library with fragment sizes between 160–230 bp, the *EGFR*-T790M frequency was increased (1.04%) when compared to the library with fragment sizes between 130–160 bp (0.30%; **Figure 7B**).

## Discussion

This study showed that a consistent fragment length difference occurs when comparing ctDNAs and normal cfDNA, with the mutant allele almost always associated with a shorter ctDNA fragment size, which is consistent with previous findings (Jiang et al., 2015; Underhill et al., 2016; Mouliere et al., 2018). However, some mutant ctDNAs were found to have a longer fragment size when compared to normal cfDNAs and were associated with a low mutant allele frequency, which has not been previously reported. Furthermore, this study showed that in cancer patient plasma samples, the ctDNA fragment length is associated with the mutant allele frequency and may even be affected by it.

Here, blood samples were obtained from 105 patients that contained different cancer driver gene mutations, such as NSCLC patients with an *EGFR* gene mutation and colorectal cancer patients with a *BRAF* mutation. In general, mutant ctDNA fragments were found to be much shorter than normal cfDNA fragments regardless of the histological type or driver gene mutation. However, ctDNA fragments with a low mutant allele frequency were found to be longer than normal cfDNA fragments. In another study, longer mutant ctDNA fragments were also detected in cancer patient blood samples, but this phenomenon could not be explained at the time (Mouliere et al., 2018). The findings presented herein may partially explain the origin of these longer mutant ctDNA fragments.

In a previous study examining HCC plasma samples, ctDNAs with low fractional concentrations were also found to have a longer size distribution relative to the healthy controls (Jiang et al., 2015), which is similar to the observations in this study. However, the previous study only compared fragment length differences between cancer patients and healthy donors, and did not distinguish mutant ctDNA fragments from normal cfDNAs due to experimental design limitations. Taken together, these findings could suggest that early-stage tumors tend to release longer ctDNA fragments at a low-frequency, but this hypothesis requires further examination.

Mutant ctDNA fragments with a low allele frequency are hard to be accurately detected. Here, two advanced technologies to detect mutant ctDNA fragments and monitor mutant allele frequency were employed to overcome this obstacle. The cfDNA fragment sizes were accurately determined using deep-sequencing technologies, and the mutant allele frequencies were further confirmed using ddPCR. However, even these advanced technologies are susceptible to false positives.

Furthermore, the lost enrichment phenomenon of short fragments observed in this study may be related to factors such as the designed probe size (120 bp), cfDNA purification, and library construction. Moreover, the findings presented herein indicate that size selection can further improve the ctDNA detection rate and accuracy. Additionally, it would seem that when constructing a ctDNA library for early-stage cancer patients, a larger DNA fragment size (> 167 bp) should be enriched, while in later stages, enrichment of shorter DNA fragment size (< 167bp) is more beneficial.

In summary, this study demonstrates that plasma ctDNAs are generally shorter than normal cfDNAs. However, for cancer patients with a low mutant allele frequency or early tumor stage, mutant ctDNA fragments are longer than normal cfDNAs. These findings may potentially facilitate the accurate detection of cancer gene mutations when utilizing liquid biopsies, and improve the application of ctDNA detection in early cancer diagnoses.

## Data Availability Statement

FASTQ data files for this study can be found in the NCBI Sequence Read Archive (SRA) database (BioProject ID: PRJNA562379).

## Ethics Statement

Our research was approved by the Medical Research Ethics Committee of Chifeng Municipal Hospital (Ethics [2018] No. 017). All patients provided informed written consent before de-identification.

## Author Contributions

GT, JL, and LZ designed the project and analyzed the data. XL, WTW, and JL wrote the manuscript. SL, YW, LP, YH, SY, GZ, SX, and HP collected the data. CQ, LS, and KZ did the ddPCR experiments. WWW, DY, YP, QL, and PB modified and reviewed the manuscript.

## Funding

This research was funded by the Natural Science Foundation of China (Grant 61803151), the Chifeng Municipal Hospital In-Situ Science and Technology Plan Project (2017-KY012), the Natural Science Foundation of Hunan province (Grant 2018JJ3570) and the Project of Scientific Research Fund of Hunan Provincial Education Department (Grant 17A052).

## Conflict of Interest

Authors JL, WTW, YH, CQ, LS, SY, KZ, GZ, HP, QL, YP, WWW, DY, SX and GT were employed by the company Geneis (Beijing) Co. Ltd.

The remaining authors declare that the research was conducted in the absence of any commercial or financial relationships that could be construed as a potential conflict of interest.
